# Developing an animal-assisted support program for healthcare employees

**DOI:** 10.1186/s12913-020-05586-8

**Published:** 2020-08-03

**Authors:** Bella Etingen, Rachael N. Martinez, Bridget M. Smith, Timothy P. Hogan, Laura Miller, Karen L. Saban, Dawn Irvin, Becky Jankowski, Frances M. Weaver

**Affiliations:** 1 Center of Innovation for Complex Chronic Healthcare (CINCCH), Edward Hines Jr. VA Hospital, 5000 South 5th Avenue (151H), Hines, IL 60141 USA; 2 Northwestern University Feinberg School of Medicine, Chicago, IL USA; 3 Center for Healthcare Organization and Implementation Research (CHOIR), Edith Nourse Rogers Memorial Veterans Hospital, Bedford, MA USA; 4 Department of Population and Data Sciences, UT Southwestern Medical Center, Dallas, TX USA; 5 Women’s Mental Health, Mental Health Service Line, Edward Hines Jr. VA Hospital, Hines, IL USA; 6 Marcella Niehoff School of Nursing, Loyola University Chicago, Maywood, IL USA; 7 PAWSitive Therapy Troupe, Chicago, IL USA; 8 Parkinson School of Health Sciences and Public Health, Loyola University Chicago, Maywood, IL USA

**Keywords:** Animal therapy, Employee burnout, Employee wellness, Healthcare workers, Organizational behavior

## Abstract

**Background:**

Employee burnout and its associated consequences is a significant problem in the healthcare workforce. Workplace animal therapy programs offer a potential strategy for improving employee well-being; however, research on animal therapy programs for healthcare workers is lacking. This study aimed to evaluate the feasibility, acceptability and preliminary impact of an animal-assisted support program to improve healthcare employee well-being.

**Methods:**

In this mixed-methods pilot intervention study, we implemented an animal-assisted support program in a multidisciplinary healthcare clinic at a large VA hospital. The program included 20 sessions over 3 months, each approximately 1-h long. Real-time mood data were collected from participants immediately before and after each session. Participation rates were tracked in real time and self-reported at follow-up. Data on burnout and employee perceptions of the program were collected upon completion via a survey and semi-structured interviews. Differences in mood and burnout pre/post program participation were assessed with t-tests.

**Results:**

Participation was high; about 51% of clinic employees (*n* = 39) participated in any given session, averaging participation in 9/20 sessions. Mood (on a scale of 1 = worst to 5 = best mood) significantly improved from immediately before employees interacted with therapy dogs (*M* = 2.9) to immediately after (*M* = 4.5) (*p* = 0.000). Employees reported significantly lower levels of patient-related burnout (e.g., how much exhaustion at work relates to interaction with patients) after (*M* = 18.0 vs. before, *M* = 40.0) participating (*p* = 0.002). Qualitative findings suggested that employees were highly satisfied with the program, noticed an improved clinic atmosphere, and experienced a reduction in stress and boost in mood.

**Conclusions:**

Establishing an animal-assisted support program for employees in a busy healthcare clinic is feasible and acceptable. Our pilot data suggest that animal-assisted programs could be a means to boost mood and decrease facets of burnout among healthcare employees.

## Background

Burnout in the workplace is characterized by depletion of employees’ emotional resources and reduction of their feelings of success and achievement [1]. Within the healthcare setting, the experience of burnout among employees is associated with a number of negative outcomes, including poor mental [2] and physical [3] health, increased absenteeism [4] and turnover intention [4, 5], diminished job satisfaction [6], and decreased healthcare quality and safety (e.g., more frequent medical errors, reduced empathy toward patients, diminished patient satisfaction) [6–15].

Evidence suggests that burnout is highly prevalent among healthcare employees, impacting more than half of healthcare providers and staff [4, 5, 16–21]. These high levels of burnout may negatively impact the quality of patient care [22, 23] and lead to increased turnover [24], which can result in resource strain for the system and disrupt care continuity for patients.

One strategy for improving morale in the workplace and protecting against the potential for burnout is offering animal-assisted support programs for employees. Evidence suggests that such programs can have significant positive impacts on employee well-being, and have been associated with reductions in workplace stress and absenteeism, and improvements in employees’ mood, health, productivity, job satisfaction, and work quality [25–28]. Animal-assisted support programs have been successfully offered in select work settings (e.g., office settings, manufacturing, sales) [25, 26]; however, there is limited literature to-date assessing the impacts of animal-assisted support on the well-being of healthcare employees working in clinical settings [29]. The objectives of this study were to evaluate the feasibility, acceptability and preliminary impacts associated with offering an animal-assisted support program for employees of a multidisciplinary healthcare clinic.

## Methods

### Data and participants

#### Design

We completed a mixed-methods intervention pilot study using an explanatory sequential approach (collecting and analyzing quantitative followed by qualitative data). The study ran from Spring 2018 to Spring 2019; animal-assisted program sessions were held in late Summer/early Fall of 2018.

#### Participants/setting

We implemented an animal-assisted support program in a multidisciplinary healthcare clinic housed within a large midwestern VA hospital. We invited all providers and staff working in that clinic (*n* = 39) to participate.

### Study design

#### Intervention/implementation strategies

To conduct the program visits for employees, we partnered with a community-based, all volunteer animal therapy organization that had an existing relationship with the hospital for hosting animal-assisted support visits with patients. Program planning was done in conjunction with clinic leadership and employees, and leadership of the animal therapy organization. We worked with clinic employees to determine program logistics, including where and when sessions would be held such that the most employees possible would have an opportunity to participate. We worked with the animal therapy organization to determine appropriate duration for the visits and availability of volunteers to conduct visits during the days and times indicated as most convenient by the clinic employees.

We held 20 program sessions over the course of 3 months. Each session was approximately 1-h long and was held in a centrally located conference room within the clinic on Monday/Friday and Tuesday/Thursday on alternating weeks, in the mid-afternoon (around lunchtime). Employees participated in the sessions as their schedule allowed; we did not standardize how long employees were able to interact with the dogs nor what activities they engaged in with the dogs (both of which were at the employee’s discretion).

We used several strategies to support program implementation. Prior to program implementation, we distributed a ‘key facts sheet’ highlighting important information about the program and an informational letter with program details and dates to all clinic employees. We placed a calendar on the door of the conference room indicating visit dates, and coordinated with the hospital’s cleaning staff to ensure they knew the room would need attention after each visit. When the program was initiated, we sent reminder emails to clinic employees and the dog handlers reminding them of sessions. During each visit, we placed additional signage outside of the conference room and at the front desk to remind employees that we were holding a visit at that time.

### Data collection

We collected data pre-, during, and post-program implementation: 

#### Pre-implementation survey

We fielded a baseline survey with clinic employees to gather information needed to refine and finalize program logistics and collect baseline data on employee outcomes (personal, work-related and patient-related burnout; interest in program participation; convenient days/times of day for participation; demographics). The survey along with an informational letter was distributed during a clinic meeting to all staff in attendance and copies were also distributed to all clinic employees after the meeting to ensure those not in attendance at the meeting had the opportunity to participate. A reminder survey was distributed 2 weeks later to optimize participation. The survey took approximately 5–10 min to complete.

#### Session participation tracking and pre/post session feedback

Participation rates were tracked in real-time by the study staff. We worked with clinic leadership to ascertain the total number of employees per shift. Participation was also self-reported on the post-implementation survey. In addition, each employee was asked to fill out a visual-analogue scale [30] indicating their current mood immediately before and after each of their interactions with the therapy dog.

#### Post-implementation survey

Follow-up data on employee outcomes (e.g., personal, work-related and patient-related burnout), program participation (e.g., whether the respondent participated, how many sessions they participated in), and employee perceptions of the program were collected upon program completion using the follow-up survey and semi-structured interviews (see below). Post-implementation survey distribution processes mirrored those used during the pre-implementation survey.

#### Semi-structured key informant interviews

Semi-structured interviews were conducted with key stakeholders (e.g., clinic employees, dog handlers) to examine perceptions of program feasibility and acceptability, and overall experiences. All clinic employees and dog handlers were invited to participate in an interview. Interviews were typically 30 min in duration, were audio recorded, and subsequently transcribed verbatim.

### Measures

The following key outcomes were assessed:

#### Participation

Observed participation rates comprised the proportion of employees who participated in each session out of the number of employees typically on that shift during the day of the week we held that session. The overall proportion of clinic employees who participated in the program and the average number of sessions employees participated in were calculated based on data provided by respondents on the post-implementation survey.

#### Real-time mood

Real-time mood was measured using a visual-analogue scale created specifically for use in this study. The scale ranged from 1 = worst mood to 5 = best mood.

#### Burnout

Burnout was measured using the Copenhagen Burnout Inventory (CBI), a valid and reliable measure of employee perceptions of burnout [31]. The CBI is comprised of 19 questions that map onto 3 types of burnout (personal, work-related, and client (i.e., patient)-related burnout). Higher scores indicate greater burnout.

#### Satisfaction and experiences

On the post-implementation survey, we asked participants to rate the extent to which they liked the program (1 = not at all to 5 = to a very large extent), and to tell us about their general experiences with the program using an open-ended, short-answer question.

#### Semi-structured key informant interviews

We asked employees to comment on program feasibility (e.g., issues related to program participation and space), acceptability (e.g., issues related to program implementation and impacts on their work experience), and general experiences with the program (e.g., perceptions of the dogs and dog handlers, how participation impacted their wellness and the general atmosphere of the clinic, their interest in participating in such a program in the future, and suggestions for improvement). We asked the dog handlers about feasibility issues, including perceptions of frequency and length of sessions and space, acceptability, including issues related to program implementation and setting, and general perceptions of the program, including how they thought the program was received by clinic employees.

### Analyses

Adoption rates, real-time mood, burnout, and employee perceptions of the program were examined using descriptive statistics. Differences in mood and burnout before and after interacting with the therapy dogs were assessed using t-tests. An alpha level of 0.05 was used to determine statistical significance.

Qualitative data from the semi-structured key informant interviews was analyzed by two qualitative experts using an inductive and deductive data-driven coding approach [32, 33] to identify key themes. An initial list of codes was created based on the major topics of interest covered by the interview guide; inductive codes were developed both within the deductive codes to reflect additional themes that arose from the data (i.e., inductive sub-codes) and separate from the deductive codes as warranted by the data. Both qualitative experts coded each transcript independently, and subsequently convened to discuss codes and resolve any discrepancies until full agreement was reached for each code. Open-ended responses on the post-implementation survey detailing respondent’s perceptions of the program were analyzed similarly.

Statistical analyses were conducted using SPSS Version 23 (IBM Corp., Armonk, New York). Qualitative analyses were conducted using NVivo Version 12 (QSR International Pty Ltd.). This study was approved by the appropriate VA Institutional Review Boards.

## Results

We received completed pre-participation mood scales following approximately 91% of employee encounters with the animal-assisted support program and post-participation mood scales from approximately 86% of encounters. Twenty-two completed pre-implementation surveys (56.4% response rate) and 16 completed post-implementation surveys (41.0% response rate) were returned. We completed 10 key informant interviews (five with clinic employees and five with dog handlers). A total of 12 different dog handlers conducted visits for the study.

Survey participants were predominantly female, of white race and non-Hispanic ethnicity, and included physicians, nurses, individuals with other clinical responsibilities, and non-clinical support staff (Table 1). Interview participants were predominantly female, and represented a range of position types (e.g., physicians, nurses, individuals with other clinical responsibilities, non-clinical support staff). Survey, mood scale, and adoption tracking results are presented below. Qualitative findings from the semi-structured interviews are included where appropriate, to offer additional insights. Quotes are attributed to an “employee” generally to ensure anonymity among the small sample of participants.

**Table 1:** Survey participant demographics

	Pre-Implementation Survey (***n*** = 22)	Post-Implementation Survey (***n*** = 16)
**Female Gender**	100% (*n* = 17)	100% (*n* = 15)
**Race**		
White	45.5% (*n* = 10)	37.5% (*n* = 6)
African American/Asian/American Indian/Alaska Native	27.3% (*n* = 6)	31.3% (*n* = 5)
Don’t Know/Not Sure/Would Rather Not Say	18.2% (*n* = 4)	31.3% (*n* = 5)
**Non-Hispanic Ethnicity**	90.0% (*n* = 18)	81.3% (*n* = 13)
**Age**		
49 or Younger	65.0% (*n* = 13)	50.0% (*n* = 8)
50 or Older	35.0% (*n* = 7)	50.0% (*n* = 8)
**Role In VA**		
Attending Physician/Nurse Practitioner	42.9% (*n* = 9)	26.7% (*n* = 4)
Nurse	28.6% (*n* = 6)	33.3% (*n* = 5)
Other Clinician/Non-Clinical Support Staff	28.6% (*n* = 6)	40.0% (*n* = 6)

### Implementation processes

#### Participation

**Fig. 1:**
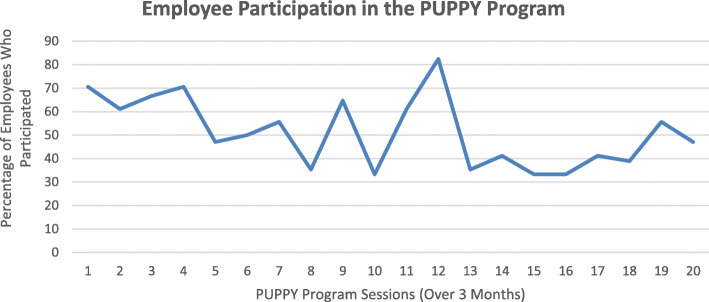
Distribution of employee participation in program sessions

Participation in the program was high; on any given day we held a session, about 51% (range: 33–82%) of the employees working in the clinic (*n* = 39) participated (Fig. 1), and survey responses indicated that employees (*n* = 15) participated in 9 of the 20 visits on average (range: 1–18).

Interviews suggested that overall, employees were satisfied with the number of visits, when visits were held, and how long the therapy dogs were present during the visits. The program scheduling gave them opportunities to participate on days/times that their clinic schedule allowed. The length of each session provided a window of time that the employees could visit with the dogs, if even for a few minutes. Some employees expressed that they wanted to participate more often and/or spend more time with the dogs, but were sometimes unable to because of the busyness of the clinic.

#### Facilitators and challenges for implementation

The key informant interviews revealed a number of factors that influenced employee perceptions about program implementation. Interviewees reported that the space used for the visits was somewhat small, but it was the best option for this clinic on the floor where their employees provide patient care. Additionally, employees indicated that the proximity of the room enhanced program accessibility by allowing them to jump in to interact with the dog amid patient care/other clinic responsibilities, which was convenient and made it easier to participate. Interviewees also noted that it was helpful to have the visits in the same room each session, and that the location was optimal given it was nearby employee workstations but easily avoidable for patients who may be leery of dogs. One employee noted that, because the room was used for many other purposes as well as the program, it was important to clean thoroughly after each visit.

Employees mentioned that seeing the program signage in highly visible areas increased awareness of when the dogs were visiting and facilitated participation. Additionally, employees reported that the kick-off meeting prior to program implementation and emails sent by the research team in advance of dog visits helped inform them of the program and when to expect the dogs in the clinic.

Dog handlers also offered insights about program implementation, providing suggestions for how to support the dogs and handlers. Elements of the program implemented by the research team that were helpful to dog handlers included email reminders before each session, detailed directions to facility, and particularly, an escort from the lobby of the hospital to the clinic conference room, which dog handlers noted made them feel welcomed. Dog handlers suggested that reminders about the time necessary to park and interact with curious patients and others in the hospital en-route to the clinic could also be helpful.

### Program outcomes

#### Mood (Table 2)

Participating in the program significantly improved employee’s mood in real-time (t(316) = − 17.97, *p* = 0.000), with average reported mood scores improving significantly from immediately before the employees interacted with the therapy dog (*M* = 2.9) to immediately after (*M* = 4.5). Anecdotally, interviews revealed that employees experienced a positive mood boost from spending time with the dogs, which in turn, affected how they approached their subsequent clinical responsibilities, as one employee expressed:

“. . .[the PUPPY program sessions] did definitely improve my overall mood. So if I was seeing a Veteran shortly thereafter, they would, of course, be the beneficiary of that mood.”

**Table 2:** Self-reported employee mood and burnout before vs. after program participation

*Values represent means*	Pre-Participation	Post-Participation	***p***-value
**Mood**^**a**^	**2.9**	**4.5**	**0.000**
**Patient-Related Burnout**^**b**^	**40.0**	**18.0**	**0.002**
Work-Related Burnout ^b^	53.7	48.0	0.38
Personal Burnout^b^	52.5	46.9	0.40

^a^Measured via the Puppy Mood Scale

^b^Measured via the Copenhagen Burnout Inventory (CBI) [30]

#### Burnout (Table 2)

Employees reported significantly lower levels of patient-related burnout (e.g., how much of one’s exhaustion at work relates to interaction with patients) after (*M* = 18.0) vs. before (*M* = 40.0) participating in the program (t (36)=3.33, *p* = 0.002). Differences in average reported personal (pre = 52.5 vs. post = 46.9, *p* = 0.40) and work-related (pre = 53.7 vs. post = 48.0, *p* = 0.38) burnout were not significant, but trended in the hypothesized direction. During the interviews, employees were asked to describe how the PUPPY program affected their overall work experience in the clinic. Employees felt that the program offered a therapeutic break during their workday, which reduced mid-day burnout. As described by one employee:

“. . .you might have a real busy day administratively or with patients, and obviously doing mental health as I do sometimes can be somewhat draining, and it gave you just that great recharge that you would need to kind of disconnect from your workday for a short period of time, and then that recharge that you need to go back in and start fresh and finish your day.”

Another employee described how participating in the program reduced their levels of stress over time, highlighting the importance of offering the program for multiple sessions:

“. . . I didn’t feel any effects in the beginning. But, when I was a little more intentional about going to the pet therapy, and kind of settling myself down and participating a little bit with the animal and the trainer, I really did see some benefits. . . It just took me two or three, or four sessions to really kind of get into it and feel like, let’s see if it really does make a difference. And then when I really did make a connection then I really looked forward to going. And I was like, yeah, this really does help me feel a little better. And this is for me.”

#### Satisfaction and experiences

Post-implementation survey data indicated that employee satisfaction with the program and implementation was high, with 71% of respondents (*n* = 14) reporting that they liked the program to a very large extent and another 14% to a moderate extent. Employees gave the program resounding endorsements, reporting that the benefits were far-reaching, both for the individual and the clinic as a whole. Interviewees similarly highlighted the positive interactions they had with the dogs and dog handlers, expressing enjoyment from observing and petting the dogs and chatting with the dog handlers.

Employees also noted that the program stimulated social interaction and enhanced the atmosphere in the clinic, reporting that there was “excitability among the staff” when the dogs were in the clinic. Employees mentioned that they would pop into each other’s offices on program sessions days to encourage one another to visit with the dog. From a dog handler’s perspective, the program offered an opportunity for coworkers to come together, observing that “.. .you could just tell they were relaxing, and chatting about other things, and not work.” Employees also appreciated the respectful nature of the program, noting the steps taken to ensure that having the dogs in the clinic was not intrusive nor disruptive to workflow.

In addition, qualitative findings suggest that the program had an unintended but positive indirect effect, influencing even those staff members who did not have much interaction with the dogs. For example, one employee stated in an open-ended survey response that they “enjoyed how much [their] colleagues enjoyed [the dogs]. It appeared to lift the morale on this unit.” For those who did participate, the benefits extended beyond the workplace and for some, followed them home. One employee expressed:

“I think that it definitely contributed on some level to my wellbeing or just decreasing my stress by a little, and distracting me, or giving you an exposure that. . .for some people who don’t have pets, [don't get] to have. I went home and talked about the animals, brought pictures of the animals, so it added a lovable dimension to my life. And I think that it’s got potential to be something more, when you’re really looking at that big employee wellness picture. You know, it could really be beneficial.”

Importantly, interviewees were appreciative that this program was offered specifically to employees, with the intention of improving staff well-being. One employee articulated:

“. . .it helped me to realize that the VA was trying to do something to help me. You know, because we’re always really focused on helping the Vet[erans], and making the Vet[erans] feel at home, and it was like, oh, it’s nice that they’re thinking that we need to take care of ourselves too, in order to do a good job with our Vet[eran]s.”

## Discussion

To the best of our knowledge, this is among the first published accounts of the feasibility, acceptability and preliminary impacts of using animal-assisted support within a healthcare setting to improve workforce well-being. This project provides the foundation for future research, including effectiveness trials, by showing that implementing this type of program in a busy clinic is feasible, and the program is acceptable to healthcare providers and staff. Additionally, our results strongly suggest that offering an animal-assisted support program to healthcare employees may be positively impactful on workforce morale, which may in turn aid in improving the quality of care and service they provide to patients and ultimately, patients’ experience with care.

Notably, our results suggest that animal-assisted programs could be a means to boost mood and decrease facets of burnout (specifically, patient-related burnout) among healthcare employees. This is an important finding, because research indicates that experiencing patient-related burnout is associated with decreased job performance [34] and turnover intention [35] among healthcare workers. Accordingly, improving this particular aspect of burnout in tandem with mood may facilitate employees to have more positive interactions with patients, which could improve a number of important outcomes including patient satisfaction and experiences with care.

We did not, however, find that program participation significantly impacted work-related or personal burnout among the employees in our sample. These findings may signify that participating in an animal-assisted support program impacts facets of burnout differently. It may also, however, reflect the limited scope of our feasibility pilot, including that our work was contained to one hospital clinic and the resulting small sample size limited the power of our study. Accordingly, we cannot be sure that the lack of significant differences in two of our burnout sub-scales was attributable to the intervention not meaningfully impacting those aspects of burnout or a lack of power to detect differences.

Historically, interventions designed to improve workplace burnout have focused on the individual (e.g., bolstering individuals’ resiliency, strategies for personal behavior change) [36, 37]. However, literature suggests that organizational-level strategies may be preferable to and more impactful than these individually focused methods [36], signaling that intervention efforts should focus on programs that make the work environment less stressful and more primed toward positive employee interactions and experiences. While the animal-assisted support program that we describe here does not ameliorate some organizational factors that lead to burnout (e.g., staffing, workload), other aspects make it exactly that – an organizational resource poised to improve the employee experience and optimize the organizational climate of their unit. Importantly, the program allows for flexible participation that can be adapted to most any employee’s schedule.

Given the success of the program in this study, the implementation process we used provides a solid foundation for future efforts to implement employee-targeted animal-assisted support programs in healthcare settings. We found several strategies particularly helpful to our implementation efforts, including pre-implementation engagement of end-users, providing ample information about the program to employees before and during implementation, and exercising flexibility with logistics. However, it is important to note that implementation of this type of program is not one-size-fits-all. The scheduling and space protocols we describe were specifically developed to reflect the clinic context in which we were working. Each healthcare facility that embarks upon such a program should coordinate with each of its clinics to determine their preferences for scheduling and space, and to ensure that their employees can participate in program sessions. One possibility for clinics that do not have space available to dedicate for the program is to make rounds with the dog to people’s offices or hold the visits in more public spaces in the hospital, such as lobbies.

Our results also highlight that employee perceptions of such programs may evolve favorably over time and with exposure, highlighting the importance of offering multiple program sessions over time. In addition, it is likely that, were a program like this to be discontinued after some time, its positive impacts would wear off, further substantiating the need to offer program sessions on an ongoing basis so its desired impacts have the opportunity to be realized. One key question, which was beyond the scope of the current study, is how many times (and how regularly) individuals would need to participate in the program to reap benefit from it, as well as how long individuals need to interact with the dogs during any given session and whether there are certain activities that participants can do during these interactions that would be more impactful. Future research should examine these important questions, including what the right “dose” of such a program is for it to have optimal effects and how long the benefits of participation last.

In order to optimize future program implementation and facilitate larger scale roll-out, additional research is needed to assess factors that influence program implementation in various healthcare clinics across facilities of varying size and complexity. Future work might also explore the feasibility of implementing employee-facing animal therapy programs in facilities where these programs do not currently exist for patients, as well as the factors that are associated with why the program may positively impact facets of employee wellness and (as mentioned above) what the right dose of the program is to optimize its impact.

### Limitations

The generalizability of our results may be limited as a result of recall/social desirability bias of self-report visual-analogue scale and survey data, as well as some factors related to the composition of the employees working in the clinic (i.e., female gender predominance), and that clinics may differ in baseline satisfaction and cohesiveness. In addition, we did not validate the visual analogue scale used, including its appropriateness for a healthcare worker population, and the utility of a 5-point scale to capture mood (e.g., as opposed to a 10-point scale). Moreover, the way that workflow and staffing is structured in this clinic may impact generalizability of the implementation-related information to other similar settings wherein staffing is handled differently. Because not everyone likes dogs, this program may not be appropriate for all employees; alternative strategies to enhance employee wellness should be considered for individuals who do not wish to interact with a dog. Of note, because of the quasi-experimental design used and the nature of our pre/post data, the analyses presented were descriptive and as such, we cannot draw causal inferences from them.

## Conclusion(s)

Our data suggest that animal-assisted programs could be a means to boost mood and decrease facets of burnout among healthcare employees. The study further suggests that establishing such a program for employees in a busy healthcare clinic is feasible and acceptable to employees. Based on these results, we believe that additional research to establish the effectiveness of animal-assisted support in improving healthcare employee wellness is warranted. In particular, randomized controlled trials are needed to systematically assess the impacts of this type of program on healthcare employee outcomes and identify factors that can influence their implementation.

## Data Availability

The datasets generated and/or analyzed during the current study are not publicly available due to institutional restrictions.

## Abbreviations

CBI: Copenhagen Burnout Inventory
VA: US Department of Veterans Affairs
